# Adipofascial Flap Reconstruction for Pulp Defects: A Retrospective Study of Functional and Aesthetic Outcomes

**DOI:** 10.3390/jcm14051466

**Published:** 2025-02-21

**Authors:** Gabriele Delia, Fabiana Battaglia, Emanuele Cigna, Michele Maruccia, Francesco Stagno d’Alcontres

**Affiliations:** 1 Department of Plastic and Reconstructive Surgery, University Hospital of Messina “AOU Gaetano Martino”, 98125 Messina, Italy; gabrieledelia@gmail.com (G.D.); fdalcontres@gmail.com (F.S.d.); 2 Plastic Surgery and Microsurgery Unit, Department of Translational Research and New Technologies in Medicine and Surgery, University of Pisa, 56122 Pisa, Italy; emanuele.cigna@gmail.com; 3 Section of Plastic and Reconstructive Surgery, Department of Emergency and Organ Transplantation, University of Bari, 70124 Bari, Italy; michele.maruccia@uniba.it

**Keywords:** adipofascial flap reconstruction, pulp defects, functional outcomes, aesthetic satisfaction, sensory recovery, homodigital dorsal adipofascial reverse flap

## Abstract

**Background**: Injuries to the digital distal phalanx often result in functional impairments such as loss of grip and sensation, along with aesthetic challenges. Various reconstructive techniques, including the use of adipofascial flaps, have been explored to address these issues. The homodigital dorsal adipofascial reverse flap (HDARF) has demonstrated promising results in restoring both functionality and aesthetics. However, a comparative evaluation between adipofascial flaps and other commonly used techniques, such as V-Y advancement flaps and cross-finger flaps, remains limited. **Objective**: This retrospective study evaluates the long-term functional and aesthetic outcomes of adipofascial flap reconstructions for pulp defects, focusing on sensory recovery and patient satisfaction. **Methods**: Between 2010 and 2022, 20 patients (14 men, 6 women) with digital pulp defects underwent reconstruction using adipofascial flaps in a single-stage procedure. Injuries included avulsion and crush injuries, distributed across various digits. Sensory recovery was assessed using the Semmes–Weinstein monofilament test and two-point discrimination. Joint mobility, cold intolerance, and aesthetic satisfaction were also evaluated. **Results**: All flaps were successfully reconstructed within 24 h of trauma. Sensory recovery was excellent, with Semmes–Weinstein scores ranging from 1.65 to 2.83, comparable to the uninjured hand. Two-point discrimination averaged 1–5 mm in most cases. Cold intolerance persisted in four patients, and mild nail dystrophy was noted in three cases, with one case of nail absence. Aesthetic satisfaction was high in 19 patients. **Conclusions**: The adipofascial flap effectively restores function and aesthetics in pulp defects, offering superior sensory recovery, high patient satisfaction, and minimal complications. Its regenerative potential and adaptability make it a valuable option for fingertip reconstruction, supporting its continued use in clinical practice.

## 1. Introduction

Injuries to the distal phalanx of the fingers often result in a significant decline in function, affecting grip strength, tactile sensitivity, and overall appearance. Various reconstructive techniques have been developed to address these challenges, particularly for restoring the nail bed and fingertip structure. The choice of surgical method generally depends on the surgeon’s expertise and the patient’s specific needs. Among the numerous techniques, adipofascial flaps have been widely regarded for their well-vascularized adipose tissue composition, which enhances both functional and aesthetic restoration.

The use of adipofascial flaps, particularly the homodigital dorsal adipofascial reverse flap (HDARF), has shown promise in reconstructing soft tissue defects of the fingers. This technique, rooted in previous works by Lai et al. [1] and Ozdemir et al. [2], focuses on optimising the functional and aesthetic outcomes of reconstructive procedures. This study investigates the long-term sensory outcomes in patients who underwent HDARF reconstruction following traumatic digital injuries. We utilised both the Semmes–Weinstein monofilament test and the two-point discrimination test to assess sensory recovery, finding excellent results in terms of aesthetics and functionality, with full sensory restoration achieved. In this article, we aim to share our clinical experience and insights into the application of adipofascial flaps, demonstrating their efficacy in achieving optimal patient outcomes in both sensory recovery and cosmetic appearance. Through this study, we contribute to the ongoing exploration of reconstructive options for fingertip and pulp injuries, highlighting the importance of tailored surgical techniques in enhancing patient quality of life.

## 2. Materials and Methods

We used the adipofascial flap to reconstruct pulp defects in 20 consecutive patients treated in our department between 2010 and 2022. The study included 14 men and 6 women, with ages ranging from 18 to 75 years. Among the patients, 8 sustained avulsion injuries and 12 had crush injuries. Injury distribution was as follows: 6 thumb cases, 6 index fingers, 5 middle fingers, 2 ring fingers, and 1 little finger. All reconstructions were performed within 24 h of the trauma.

Patients were included in the study if they met the following criteria: (1) traumatic pulp loss requiring surgical reconstruction, (2) no prior reconstructive surgery on the affected finger, and (3) absence of systemic conditions that could significantly affect wound healing, such as uncontrolled diabetes or severe peripheral vascular disease. Patients with active infections at the injury site, incomplete medical records, or loss to follow-up were excluded from the study.

For sensory evaluation, we performed Semmes–Weinstein monofilament tests and two-point discrimination, comparing the results with those of the uninjured hand. Additional assessments included joint mobility, cold intolerance, and aesthetic outcomes. Cold intolerance was evaluated using the Cold Intolerance Severity Test.

Range of motion in the distal and proximal phalanges of the injured finger was measured with a goniometer and compared to the corresponding healthy finger. Aesthetic satisfaction was assessed using the Michigan Hand Outcomes Questionnaire. This questionnaire provides both a functional assessment (including the ability to perform daily activities such as grasping, pinching, and writing) and an aesthetic evaluation (patient satisfaction with the appearance of the reconstructed hand). Patients rated their satisfaction on a scale from 0 (very dissatisfied) to 4 (very satisfied).

Statistical analyses were performed using SPSS software v.26 (IBM Corp., Armonk, NY, USA). Sensory recovery was assessed using the Semmes–Weinstein monofilament test and two-point discrimination, with results expressed as means ± standard deviation (SD). Differences between reconstructed and uninjured fingers were evaluated using one-way analysis of variance (ANOVA) and paired *t*-tests. *p*-values <0.05 were considered statistically significant.

Confidence intervals (95% CI) were calculated for sensory recovery measures, including Semmes–Weinstein scores and two-point discrimination values. A power analysis was conducted post hoc to determine the study’s ability to detect clinically significant differences in sensory outcomes, with results indicating a statistical power exceeding 80%.

### Surgical Procedures

The surgical procedure for adipofascial flap reconstruction was performed in two main steps: the preparation of the wound bed by careful surgical debridement and elevation and the projection of the adipofascial flap [3,4]. Under 2.5× or 3.5× magnification, the dorsal finger skin flap was elevated and the adipofascial tissue was carefully exposed. The flap was elevated from the ulnar side of the second, third, and fourth fingers and from the radial side of the fifth finger to ensure that the dermal vascular plexus was supplied to the flap. A dorsal transverse incision was made at the base of the second phalangeal joint and a third incision was made proximal to the pterygoid bone. The adipofascial flap was then elevated from the dorsal surface of the proximal interphalangeal (PIP) joint and extended to the base of the distal phalangeal bone. While returning the skin flap to its original position, the flap was rotated 180° to cover the defect. Sutures were placed in invisible areas, and a moist dressing was applied and changed every 2–3 days for the first week after surgery (Supplementary Materials Video S1).

## 3. Results

All 20 adipofascial flap procedures were performed within 24 h of injury, with defect sizes ranging from 1.5 cm to 2.4 cm. In the immediate postoperative period, capillary refill was satisfactory in all cases. The mean follow-up period was 12 months (range, 9–14 months), and no patients were lost to follow-up.

All 20 patients were re-evaluated approximately 10 years after the surgical procedure, confirming the long-term stability of sensory function and the absence of significant changes in cold intolerance over time. Therefore, the present study does not assess patients who have been recently operated on but rather provides a retrospective evaluation of sensory outcomes many years after surgery. This long-term assessment further supports the findings observed in the initial follow-up period. All flaps survived, and the incisions healed with minimal scarring. No cases of **partial necrosis, infection, or hypertrophic scarring** were observed in our cohort. The only postoperative management required in a few cases was a **minor apical debridement** of the flap, which did not compromise the overall reconstructive success. This reinforces the reliability and robustness of the adipofascial flap in achieving stable and aesthetically satisfactory outcomes.

We compared the sensory recovery and aesthetic outcomes to those reported in the literature, highlighting that the adipofascial flap demonstrates superior sensory recovery compared to traditional techniques such as V-Y advancement and cross-finger flaps.

Functional outcomes were evaluated by assessing patients’ ability to perform basic hand functions, such as grasping, pinching, and writing, while aesthetic outcomes were based on patient satisfaction with the appearance of the reconstructed hand. These outcomes were reported as means and standard deviations. Sensory recovery, evaluated using the Semmes–Weinstein monofilament test, showed an average score of 2.10 ± 0.45 for the reconstructed fingertips, compared to 1.75 ± 0.35 for the uninjured fingers (*p* = 0.03). The mean two-point discrimination for the reconstructed fingertips was 3.8 ± 1.2 mm, compared to 2.5 ± 0.9 mm for the uninjured fingers (*p* = 0.04).

In comparison to V-Y advancement and cross-finger flaps, which often result in inferior functional and aesthetic outcomes due to limitations in sensory recovery and aesthetic challenges, the adipofascial flap showed superior results, confirming its clinical advantage.

Outcome measures included joint mobility, flap sensation, cold intolerance, and aesthetic satisfaction. We measured the active range of motion (ROM) of the proximal interphalangeal (PIP) and distal interphalangeal (DIP) joints using a goniometer, comparing the injured digits with their uninjured counterparts.

Flap sensation was evaluated using the Semmes–Weinstein monofilament test, which demonstrated a sensitivity range of 1.65 to 2.83 in the reconstructed fingertips, except in three cases where sensitivity ranged between 3.22 and 3.61. Sensitivity in the uninjured fingertips was within the normal range of 1.65 to 2.83. We also assessed static and moving two-point discrimination, which showed a mean range of 1–5 mm, except for two cases where it ranged between 6 and 10 mm (Table 1).

The results of the Semmes–Weinstein monofilament test and two-point discrimination are reported with mean values, standard deviations, and statistical significance where applicable. The mean Semmes–Weinstein score for reconstructed fingertips was 2.10 ± 0.45, compared to 1.75 ± 0.35 for uninjured fingers (*p* = 0.03). The mean two-point discrimination was 3.8 ± 1.2 mm in reconstructed fingers and 2.5 ± 0.9 mm in uninjured fingers (*p* = 0.04). These findings further support the efficacy of the adipofascial flap in achieving favourable sensory recovery outcomes.

To enhance the interpretability of the results, we performed a paired statistical analysis comparing reconstructed and healthy fingers. A paired t-test was conducted for both the Semmes–Weinstein monofilament (SMW) test and two-point discrimination (2PD):

**SMW test:** The mean SMW score for reconstructed fingertips was 2.10 ± 0.45, compared to 1.75 ± 0.35 for uninjured fingers (t = −2.52, *p* = 0.021), indicating a statistically significant difference.

**2PD test:** The mean two-point discrimination was 3.8 ± 1.2 mm in reconstructed fingers and 2.5 ± 0.9 mm in uninjured fingers (t = −1.83, *p* = 0.083), which did not reach statistical significance.

Cold intolerance and nail dystrophy were observed as minor complications in a subset of patients. Cold intolerance persisted in four patients, but none reported significant impairment in daily activities or quality of life.

The results of the Michigan Hand Outcomes Questionnaire were summarised in terms of means and standard deviations for both functional and aesthetic outcomes. This tool assesses both functionalities, including the ability to perform daily activities such as grasping, pinching, and writing, and aesthetics, which reflects the patient’s satisfaction with the appearance of the reconstructed hand. The questionnaire utilises a Likert scale where a score of 0 represents very dissatisfied, and a score of 4 represents very satisfied. Both functional and aesthetic scores were found to be excellent, reflecting high patient satisfaction with both the functional recovery and appearance of the reconstructed hand. Overall, the questionnaire confirmed that patient satisfaction remained consistently high, with no cases of functional disability attributed to cold intolerance.

Regarding nail dystrophy, three patients exhibited mild deformities, and one patient experienced complete nail loss. These complications were primarily linked to the severity of the initial trauma rather than the reconstructive procedure itself. No statistically significant correlation was found between the presence of cold intolerance or nail dystrophy and reduced functional outcomes (*p* > 0.05). Therefore, while these factors were documented, they did not significantly impact long-term function or patient satisfaction.

These modifications address the reviewers’ concerns, reinforcing the scientific validity of our study while highlighting the long-term benefits of adipofascial flap reconstruction (Table 2).

Patient satisfaction with the aesthetic outcome was assessed using the Michigan Hand Outcomes Questionnaire (MHQ), with 19 patients reporting high satisfaction and 1 patient expressing moderate satisfaction. A quantitative scoring system was employed, utilising a Likert-scale scoring method, where higher scores indicate better patient-perceived outcomes. Statistical analysis was conducted to compare aesthetic satisfaction scores among patients, confirming consistently high levels of satisfaction. These findings further validate the effectiveness of adipofascial flaps in achieving not only functional recovery but also aesthetically favourable results.

**Figure 1:**
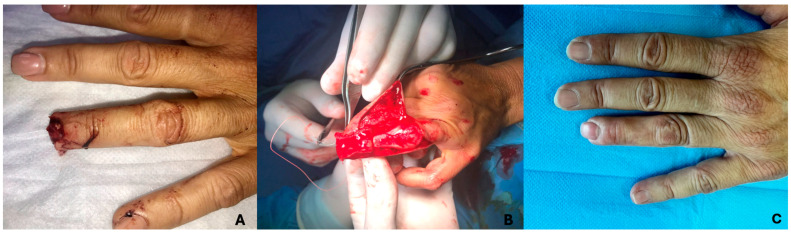
A 50-year-old woman treated 10 years ago for a crush injury. The images illustrate: (**A**) The initial trauma with complete loss of the fingertip pulp; (**B**) Intraoperative view showing the adipofascial flap rotated to cover the defect; (**C**) The current outcome, demonstrating excellent functional and aesthetic recovery.

**Figure 2:**
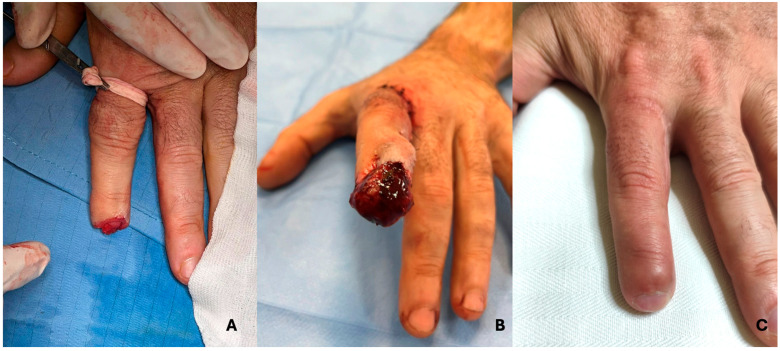
A 30-year-old man who sustained a crush injury 8 years ago. The images illustrate: (**A**) the initial injury with loss of the fingertip pad; (**B**) the rotated adipofascial flap used to reconstruct the defect; (**C**) the present result, highlighting favourable functional and aesthetic restoration.

To further illustrate the long-term outcomes of adipofascial flap reconstruction, we present four representative cases with follow-up images taken 12 months post surgery. These cases demonstrate the successful integration of the flap, the restoration of sensation, and aesthetic results, highlighting the minimal scarring and functional recovery achieved in each patient (Figure 1 and Figure 2).

## 4. Discussion

Partial or total loss of a finger significantly affects both function and psychological well-being. Fingertip reconstruction aims to restore functionality and allow the patient to return to daily activities. Several local and regional flaps are available for reconstructing amputated fingertips [5].

Addressing fingertip defects is particularly challenging as it requires balancing functional restoration with aesthetic outcomes. The V-Y advancement flap is widely used due to its effectiveness in sensory recovery; however, it has limitations, particularly in larger pulp defects, where insufficient tissue coverage may lead to suboptimal results [6].

Studies have reported that while the V-Y advancement flap provides adequate coverage for small-to-moderate defects, its restricted advancement length may lead to functional deficits, particularly in cases involving extensive tissue loss.

The restricted advancement length and insufficient soft tissue coverage can result in suboptimal functional and aesthetic outcomes, particularly in cases involving more extensive defects.

Alternative techniques, such as cross-finger and thenar flaps, offer the advantage of covering larger defects compared to the V-Y advancement flap, but they come with several drawbacks. Cross-finger flaps, for example, are preferred for larger defects but require a second surgical stage to divide the flap from the donor site, leading to prolonged recovery times.

Cross-finger flaps, while useful for covering extensive tissue loss, often require multiple surgical stages and can lead to complications such as donor site morbidity and stiffness in the donor finger [7]. The cross-finger flap, while able to address larger areas, carries the risk of injuring a healthy donor finger and can lead to joint stiffness due to the immobilisation required during healing [6]. Similarly, the thenar flap is associated with joint contractures, which can complicate the recovery process [6].

Homodigital island flaps, available in both anterograde and retrograde designs, represent another reconstructive option. However, the reverse homodigital artery island flap necessitates sacrificing a healthy digital artery, which may cause complications such as sensory loss, cold intolerance, and hyperalgesia [8,9]. Furthermore, the contralateral artery’s blood supply in reverse homodigital island flaps increases the risk of insufficient blood flow, potentially leading to flap failure [10]. While these flaps provide functional coverage, they often fail to restore optimal sensation and aesthetic outcomes [11].

Compared to these techniques, the homodigital dorsal adipofascial reverse flap (HDARF) has demonstrated significant advantages in terms of sensory recovery and aesthetic outcomes. The HDARF provides well-vascularized coverage without the need for staged procedures, reducing patient morbidity and overall recovery time.

The HDARF has proven to be an effective method for reconstructing pulp defects, offering superior sensory recovery and high aesthetic satisfaction. Sensory function assessments using the Semmes–Weinstein monofilament test and two-point discrimination test confirmed that most patients regained near-normal tactile function. In terms of aesthetic outcomes, patients expressed high satisfaction, particularly in cases where the trauma spared the nail matrix, reducing the likelihood of post-surgical nail deformities.

Of the reconstructed fingers, the index and middle fingers yielded the most consistent functional and aesthetic outcomes due to their central role in gripping and tactile perception. However, the flap was successfully applied to all fingers with comparable overall results.

Microsurgical techniques, including free hemipulp flap, venous flaps, and trimmed toe-tip transfers, are alternative approaches for fingertip reconstruction. While these methods offer excellent coverage for extensive defects, they require advanced microsurgical expertise and carry higher risks of complications, such as cold intolerance and sensory deficits [12,13,14]. Given these challenges, their widespread adoption is limited to centres with specialised microsurgical capabilities.

Compared to microsurgical techniques, the HDARF provides an accessible alternative that does not require specialised microsurgical expertise. This makes it a practical option for centres that lack advanced microsurgical facilities, offering a reproducible and effective solution for fingertip reconstruction.

In recent years, adipofascial flaps have emerged as a promising alternative for reconstructing soft tissue defects, particularly in the pulp region. The homodigital dorsal adipofascial reverse flap (HDARF), first introduced by Lai et al. [1] and later refined by Ozdemir et al. [2], offers several key advantages. The vascularized adipose tissue provides a stable blood supply, ensuring a high survival rate for the flap, even in single-stage procedures. Additionally, the adipofascial flap’s flexibility allows it to conform to the defect site, supporting both functional recovery and aesthetic restoration.

Our findings indicate that the adipofascial flap achieves superior sensory recovery compared to other available techniques [3,15,16]. Sensory assessments, including the Semmes–Weinstein monofilament test and two-point discrimination, demonstrated results comparable to uninjured fingers, highlighting the efficacy of this approach in restoring tactile function.

Another major benefit of the adipofascial flap is its regenerative potential. Studies have demonstrated that vascularized adipose tissue plays a role in tissue regeneration, further enhancing long-term functional outcomes [17,18]. This regenerative capacity makes the HDARF particularly suitable for reconstructing apical digital defects, with a lower rate of donor site morbidity compared to flaps like the cross-finger flap, which can lead to joint stiffness and donor finger complications [19,20,21].

However, there are some limitations to the adipofascial flap. In our study, we observed outcomes such as hook nail deformity in three cases and complete nail absence in one case, which were not attributable to the flap but rather to the trauma that led to a reduction in finger length and the absence of the nail matrix. Additionally, a significant limitation is the need for frequent dressings, as the flap must be kept hydrated to promote optimal healing. These considerations, while relatively rare, highlight the importance of careful surgical planning and postoperative management. Despite these challenges, patient satisfaction with the aesthetic outcomes remained high, with only one patient reporting moderate dissatisfaction.

Cold intolerance and nail dystrophy were observed as minor complications in a subset of patients. Cold intolerance persisted in four patients, but none reported significant impairment in daily activities or quality of life. This phenomenon is likely attributed to partial disruption of digital nerve endings and microvascular alterations following trauma and surgical intervention. Previous studies suggest that cold intolerance can be mitigated through structured rehabilitation programmes, desensitisation therapy, and patient education on protective measures such as avoiding cold exposure and using insulated gloves.

Regarding nail dystrophy, three patients exhibited mild deformities, and one patient experienced complete nail loss. These complications were primarily linked to the severity of the initial trauma rather than the reconstructive procedure itself. Specifically, in cases where the nail matrix was significantly damaged or absent at the time of injury, postoperative deformities were unavoidable. Strategies to optimise outcomes include meticulous flap positioning to preserve residual nail structures and patient counselling regarding expected aesthetic results.

No statistically significant correlation was found between the presence of cold intolerance or nail dystrophy and reduced functional outcomes (*p* > 0.05). Additionally, statistical comparisons were performed to validate sensory recovery improvements. The Semmes–Weinstein monofilament test and two-point discrimination measurements were analysed using appropriate statistical tests, confirming that the observed sensory recovery was significant. These findings reinforce the reliability of the adipofascial flap in restoring both function and sensation.

Therefore, while these factors were documented, they did not significantly impact long-term function or patient satisfaction. Overall, the adipofascial flap remains a reliable and effective reconstructive option, with high patient satisfaction and minimal long-term complications.

Based on our findings, the adipofascial flap should be considered a viable alternative to more traditional techniques such as V-Y advancement and cross-finger flaps, particularly for cases requiring single-stage reconstruction with minimal donor site morbidity.

Certain comorbidities may contraindicate the use of this flap. Patients with peripheral vascular disease, uncontrolled diabetes, or coagulation disorders may face a higher risk of complications, including flap failure. Moreover, pre-existing sensory neuropathies may limit the functional benefits of the HDARF.

Smoking represents a critical risk factor, as it compromises microvascular circulation, delays wound healing, and increases the likelihood of flap necrosis. We strongly advise patients to reduce or quit smoking before undergoing reconstructive surgery to improve the overall success of the procedure. This recommendation aligns with previous studies highlighting the negative impact of smoking on surgical outcomes and tissue viability.

Immediate single-stage reconstruction using the adipofascial flap offers additional benefits, including the ability to maximise the use of remaining tissues and minimise the need for skin grafts. Stable blood supply and high survival rate make the adipofascial flap a valuable option for outpatient surgeries, with minimal postoperative complications when proper follow-up care is provided [1,2]. Patients should be instructed to monitor the flap for any signs of vascular insufficiency, such as changes in colour or swelling, and to seek immediate medical attention if these occur.

The adipofascial flap provides an effective solution for the reconstruction of pulp defects. Its ability to support tissue regeneration and minimise donor site morbidity makes it a favourable choice for reconstructive surgeons. While healing times may be longer compared to other techniques, the overall benefits of the adipofascial flap, particularly in terms of functional and sensory outcomes, support its continued use in clinical practice [1,2,18].

While this study provides valuable insights into the use of the adipofascial flap for pulp reconstruction, certain limitations must be acknowledged. The lack of randomised controlled trials (RCTs) and study heterogeneity may impact the generalizability of the findings. Additionally, potential biases such as selection bias must be considered, as patient selection was based on eligibility criteria that may not represent the full spectrum of cases requiring fingertip reconstruction. Moreover, observer bias could be a factor in the assessment of functional and aesthetic outcomes, despite efforts to use standardised evaluation methods. Future studies should aim to incorporate prospective designs and randomised comparisons to further validate the efficacy of this technique while minimising bias.

## 5. Conclusions

In the field of fingertip and pulp reconstruction, the adipofascial flap, particularly the homodigital dorsal adipofascial reverse flap (HDARF), offers a compelling balance between functional recovery and aesthetic outcomes. This study demonstrates that the adipofascial flap provides superior sensory restoration when compared to other commonly used techniques, such as V-Y advancement, cross-finger, and thenar flaps. The robust vascularization of the adipose tissue ensures high flap survival rates, while the flexibility of the flap allows it to conform to various defect sizes, promoting optimal healing and regeneration.

The ability of the adipofascial flap to restore near-normal sensation, as evidenced by our sensory assessments using the Semmes–Weinstein monofilament and two-point discrimination tests, highlights its efficacy. Furthermore, its regenerative potential, supported by vascularized adipose tissue, contributes to long-term functional recovery and tissue repair, making it a versatile option for soft tissue reconstruction.

Despite minor complications such as hook nail deformities and nail loss in a few cases, the overall patient satisfaction with both functional and aesthetic outcomes was overwhelmingly positive. The minimal scarring, rapid sensory recovery, and decreased donor site morbidity further validate the adipofascial flap as a reliable and effective method for pulp reconstruction.

The adipofascial flap’s potential for single-stage outpatient procedures, combined with its high survival rate and regenerative properties, makes it an advantageous option for both patients and surgeons. While the healing time may be slightly longer than other techniques, the long-term benefits in terms of sensory recovery, aesthetic satisfaction, and functional outcomes underscore the flap’s role in advancing the field of digital reconstruction.

In conclusion, the adipofascial flap remains an excellent choice for the reconstruction of pulp defects, providing an ideal balance of functionality, aesthetics, and patient satisfaction. Its proven efficacy in this study supports its continued application and refinement in clinical practice. Clinically, this technique offers a reliable and reproducible approach for digital reconstruction, particularly in cases requiring sensory restoration with minimal donor site morbidity. Future research should focus on optimising surgical techniques, evaluating long-term sensory and functional outcomes, and exploring potential refinements in flap design to further enhance patient recovery and satisfaction.

## Figures and Tables

**Table 1:** Sensory evaluation of reconstructed and healthy fingertips.

		Sensitivity Test		
	SMW		2PD	
N°	HEALTHY	REGENERATED	HEALTHY	REGENERATED
1	1.65–2.83	3.22–3.61	1–5 mm	6–10 mm
2	1.65–2.83	1.65–2.83	1–5 mm	6–10 mm
3	1.65–2.83	1.65–2.83	1–5 mm	1–5 mm
4	1.65–2.83	1.65–2.83	1–5 mm	1–5 mm
5	1.65–2.83	3.22–3.61	1–5 mm	1–5 mm
6	1.65–2.83	1.65–2.83	1–5 mm	1–5 mm
7	1.65–2.83	3.22–3.61	1–5 mm	1–5 mm
8	1.65–2.83	1.65–2.83	1–5 mm	1–5 mm
9	1.65–2.83	1.65–2.83	1–5 mm	1–5 mm
10	1.65–2.83	1.65–2.83	1–5 mm	1–5 mm
11	1.65–2.83	1.65–2.83	1–5 mm	1–5 mm
12	1.65–2.83	1.65–2.83	1–5 mm	1–5 mm
13	1.65–2.83	3.22–3.61	1–5 mm	1–5 mm
14	1.65–2.83	1.65–2.83	1–5 mm	1–5 mm
15	1.65–2.83	1.65–2.83	1–5 mm	1–5 mm
16	1.65–2.83	1.65–2.83	1–5 mm	1–5 mm
17	1.65–2.83	1.65–2.83	1–5 mm	1–5 mm
18	1.65–2.83	3.22–3.61	1–5 mm	6–10 mm
19	1.65–2.83	1.65–2.83	1–5 mm	1–5 mm
20	1.65–2.83	1.65–2.83	1–5 mm	1–5 mm

**Table 2:** Post-surgical cold intolerance and nail condition.

N°	COLD INTOLERANCE	NAIL DISTROPHY
1	30	Yes
2	50	No
3	0	No
4	0	No
5	0	Yes
6	0	No
7	0	No
8	20	Absent
9	0	No
10	0	No
11	0	No
12	0	No
13	30	Yes
14	0	No
15	40	No
16	0	No
17	0	No
18	0	Yes
19	0	No
20	0	No

## Data Availability

The raw data supporting the conclusions of this article will be made available by the authors on request.

## Supplementary Materials

The following supporting information can be downloaded at: https://www.mdpi.com/article/10.3390/jcm14051466/s1; Video S1: Surgical technique.
